# Plasma fatty acid-binding protein 4 (FABP4) level is associated with abnormal QTc interval in patients with stable angina and chronic kidney disease

**DOI:** 10.1186/s12872-019-1134-z

**Published:** 2019-06-24

**Authors:** Chao-Ping Wang, Chia-Chang Hsu, Wei-Chin Hung, Teng-Hung Yu, Cheng-Ching Wu, I-Ting Tsai, Wei-Hua Tang, Fu-Mei Chung, Jer-Yiing Houng, Yau-Jiunn Lee, Yung-Chuan Lu

**Affiliations:** 1 Division of Cardiology, I-Shou University, Kaohsiung, 82445 Taiwan; 2 Division of Gastroenterology and Hepatology, I-Shou University, Kaohsiung, 82445 Taiwan; 3 Division of Endocrinology and Metabolism, Department of Internal Medicine, E-Da Hospital, I-Shou University, No. 1, Yi-Da Rd, Jiau-Shu Village, Yan-Chao District, Kaohsiung, 82445 Taiwan; 4 Department of Emergency, E-Da Hospital, I-Shou University, Kaohsiung, 82445 Taiwan; 5 Division of Cardiology, Department of Internal Medicine, National Yang-Ming University Hospital, Yilan, 26058 Taiwan; 6 School of Medicine for International Students, College of Medicine, I-Shou University, Kaohsiung, 82445 Taiwan; 7 Department of Nutrition, Institute of Biotechnology and Chemical Engineering, College of Medicine, I-Shou University, Kaohsiung, 82445 Taiwan; 8 Lee’s Endocrinologic Clinic, Pingtung, 90000 Taiwan

**Keywords:** Fatty acid-binding protein 4, Stable angina, Chronic kidney disease, QTc interval

## Abstract

**Background:**

Fatty acid-binding protein 4 (FABP4) (also known as adipocyte FABP or adipocyte P2) is expressed in adipocytes, macrophages, and capillary endothelial cells. Previous studies have shown associations among plasma FABP4, insulin resistance, metabolic syndrome, diabetes mellitus, greater coronary plaque burden, coronary artery disease, heart failure, and mortality. However, little is known about the relationship between FABP4 level and prolonged QT interval. The aim of this study was to investigate whether plasma FABP4 level is associated with a prolonged QT interval by analyzing 12-lead electrocardiograms (ECGs) in patients with stable angina and chronic kidney disease (CKD).

**Methods:**

This study included 397 consecutive patients with stable angina and CKD who were enrolled in a disease management program. Plasma FABP4 concentrations were measured using enzyme-linked immunosorbent assays. A 12-lead ECG recording was obtained from each patient. We assessed the relationships between FABP4 levels (both as a continuous variable and stratified by tertile) at admission and corrected QT (QTc) prolongation.

**Results:**

Patients with an abnormal QTc interval had higher median plasma FABP4 levels than those with borderline and normal QTc intervals (15.9 ng/mL vs. 10.2 ng/mL vs. 8.5 ng/mL, respectively, *P* < 0.0001). Statistically significant associations were observed between plasma FABP4 levels and QTc interval (β = 0.267, *P* < 0.0001). Using multivariate and trend analyses, a higher concentration of plasma FABP4 level was independently associated with QTc prolongation in patients with stable angina and CKD.

**Conclusion:**

In this study, plasma FABP4 levels were significantly higher in the patients with an abnormal QTc interval and were correlated with QTc prolongation. Further studies are required to elucidate whether plasma FABP4 plays a role in the pathogenesis of QTc prolongation.

## Background

The QT interval on surface electrocardiograms (ECGs) represents the time from onset of ventricular depolarization to completion of repolarization, and several studies of older adults and patients with myocardial ischemia or infarctions have reported an association between prolongation of this interval and ventricular arrhythmias that could trigger ventricular fibrillation and sudden cardiac death [1–3]. Furthermore, prolonged heart rate-corrected QT (QTc) interval has also been associated with an increased risk of coronary heart disease and cardiovascular disease mortality in the general population [4, 5]. A prolonged QTc interval is a complex trait which can be affected by both genetic and environmental factors (e.g. age and sex) [6, 7]. In addition, QTc prolongation is very common and is associated with several risk factors in patients with chronic kidney disease (CKD) [8]. CKD is associated with elevated inflammatory markers and increased with progression of renal failure [9]. A previous study suggested that elevated inflammatory mediators and activation of the renin-angiotensin system contribute to arterial calcification and vascular atherosclerosis through enhanced production of reactive oxygen species in patients with CKD [10]. Previous studies have also demonstrated that during reperfusion of ischemic myocardium, cytokines such as platelet-activating factor generated by activated neutrophils can cause some arrhythmias [11, 12]. In addition, Chung et al. showed that elevated C-reactive protein (CRP) may reflect an inflammatory state that promotes the persistence of atrial fibrillation [13]. Hence, increased levels of inflammatory mediators in patients with CKD may provoke cardiac fibrosis, vascular damage, sympathetic overactivity, and ion channel malfunction [9–14], resulting in a higher risk of cardiac arrhythmias and death.

Fatty acid-binding protein 4 (FABP4) is an adipokine produced by adipose tissues. It is a carrier protein which has been shown to carry lipophilic compounds including fatty acids between intra- and extra-cellular membranes [15, 16]. In addition, it has been shown to affect inflammation, insulin resistance, thrombogenicity, and other metabolic pathways [17–19]. A previous study suggested that FABP4 may have a negative inotropic effect on cardiomyocytes [20]. Furthermore, the expression of FABP4 in adipocytes has been positively associated with mortality [21], incident diabetes mellitus [22], greater coronary plaque burden [23], coronary artery disease (CAD) [24], and heart failure (HF) [25]. Moreover, FABP4 plasma concentrations have been reported to potentially be an early clinical marker of renal function derangement in patients with type 2 diabetes [26]. Furthermore, in isolated rat cardiomyocytes, FABP4 was shown to acutely depress shortening amplitude and intracellular systolic peak Ca (2+) in a dose-response fashion [20]. This suggests that FABP4 may play an important role in cardiac depolarization and possibly cardiac arrhythmias. Given the association between QT interval prolongation, CKD, and inflammation, and given the inflammatory mediator effects of FABP4 and its relationship to cardiovascular disease and cardiac arrhythmias, we hypothesized that FABP4 may be independently associated with QTc interval prolongation in humans.

In addition, plasma FABP4 level has been positively associated with coronary plaque burden, CAD, HF, and mortality [21, 23–25]. These observations suggest that FABP4 may play a role in the development of cardiovascular damage, and it is reasonable to assume that plasma FABP4 may be associated with conductive system injury. However, to the best of our knowledge, little is known about the relationship between FABP4 level and cardiac electro-pathology. Therefore, the aim of this study was to investigate whether FABP4 levels were associated with a prolonged QTc interval in a cohort of patients with stable angina and CKD.

## Methods

### Study population

From June 2006 to June 2015, 397 consecutive consenting patients with a clinical diagnosis of stable angina underwent angiography for the first time at the Cardiovascular Clinic of E-Da Hospital. The estimated glomerular filtration rate (eGFR) was calculated for each patient using the CKD-EPI two-level race equation within 3 to 6 months of admission [27]. Patients with CKD stage II-IV were eligible for inclusion into this study, and their baseline clinical characteristics were recorded. Stable angina pectoris was defined as retrosternal chest discomfort, precipitation by exertion, prompt relief within 30 s to 10 min with rest or nitroglycerine, and chest pain related to effort with no evidence of recent deterioration or rest pain in the past 6 months. The exclusion criteria were patients with inflammatory diseases (including sepsis or infection), collagen diseases, liver diseases, malignancy, steroid use, a bundle branch block pattern, and a history of psychosis. In addition, patients taking medications which may have influenced the QT interval including psychotropic medications and class I (e.g. flecainide, quinidine, mexiletine, and procainamide) and class III (e.g. dronedarone, amiodarone, and vernakalant) anti-arrhythmic medications were also excluded from the study. Written informed consent was obtained from each patients before entry into this study, which was approved by the Human Research Ethics Committee at our institution.

We used online software (https://www.anzmtg.org/stats/PowerCalculator/PowerANOVA) to calculate the required sample size. When the number of groups was set at three, the minimum acceptable power level was 0.80. Furthermore, the effect size was set at a medium effect size (0.25) and the significance level was set at 0.05. The results showed a sample size of 52.4 per group. The formula used to calculate the effect size was as follows:

$$ {\upeta}^2=\frac{\mathrm{Sum}\ \mathrm{of}\ \mathrm{squares}}{\mathrm{Total}\ \mathrm{sum}\ \mathrm{of}\ \mathrm{squares}} $$

Before the coronary angiography examination, the detailed records of each patient with regards to medical and personal history were reviewed. The following cardiovascular disease risk factors were assessed. Smoking status was classified as non-smoker, former smoker (having stopped smoking for ≥1 year), or current smoker. Current and former smokers were grouped for analysis and compared to the never smokers. Patients with a current or prior diagnosis of type 2 diabetes and those receiving medical therapy for type 2 diabetes were defined as having type 2 diabetes in accordance with World Health Organization guidelines [28]. Hypertension was defined as persistent elevation of systolic blood pressure (SBP) (≥140 mmHg) and/or diastolic blood pressure (DBP) (≥90 mmHg). Patients who were receiving antihypertensive therapy were also defined as having hypertension. The following were used to define hyperlipidemia according to the Adult Treatment Panel III criteria [29]: high-density lipoprotein cholesterol (HDL-C) levels of < 35 mg/dL and < 39 mg/dL for men and women, respectively; low-density lipoprotein cholesterol (LDL-C) level of ≥130 mg/dL; total cholesterol level of ≥200 mg/dL; triglyceride (TG) level of ≥150 mg/dL; or currently receiving antidyslipidemic medications.

### Laboratory measurements

Before the coronary angiography examination, plasma biochemical parameters were measured in all of the patients after fasting for 8 h. All biochemical analyses were performed at the E-Da Hospital laboratory within 2 h of the blood samples being drawn. A parallel, multichannel analyzer (Hitachi 7170A, Hitachi Ltd., Tokyo, Japan) was used to measure complete blood cell count, lipid profile (including plasma TGs, total cholesterol, LDL-C, and HDL-C), and levels of uric acid, albumin, glucose, sodium, calcium, potassium and serum creatinine. In addition, the concentrations of plasma FABP4 concentration was determined using an enzyme-linked immunosorbent assay kits (R&D Systems Inc., Minneapolis, MN, USA). The dilution and standard curves were parallel, and the intra- and inter-assay coefficients of variation of the assay were 3.4 to 5.8% (*n* = 3) and 3.1 to 6.2% (*n* = 4), respectively. A high-sensitivity method was used to measure levels of plasma CRP with an IMMAGE system (Beckman Coulter, Immunochemistry Systems, Brea, CA, USA) that had a detection limit of 0.2 mg/L. The intra-assay coefficient of variation was 4.2 to 8.7% for high-sensitivity (hs)-CRP. Samples were assessed in duplicate in a single experiment.

### Angiographic definitions

Coronary angiography was performed following standard techniques, and the degree of stenosis was assessed using quantitative coronary angiography (QCA). A minimum of two experienced interventional cardiologists who were blinded to the clinical information and serologic parameters of the patients evaluated the angiographic data. Two scoring systems were used to classify the results. The first referred to the total number of diseased vessels, in which 1-, 2-, or 3-vessel disease was defined as a > 75% reduction in the internal diameter, and a stenotic diameter of the left main coronary artery not exceeding 50%. The second scoring system was the modified Gensini scoring system, in which each coronary segment is scored depending on the importance and size of the vessel, with scores ranging from 0.5 to 5.0. For example, 0–25% stenosis of the coronary artery lumen is scored 2, 26–50% is scored 4, 51–75% is scored 8, 76–90% is scored 16, 91–99% is scored 32, and 100% is scored 64. The modified Gensini index is then calculated as the sum of the total scores for each segment [30, 31].

### Electrocardiogram, QT and QTc interval measurements

A minimum of two cardiologists who were blinded to this study manually measured the QT intervals. Twelve-lead ECGs were recorded using a standardized protocol upon enrollment into the study. The QTc of lead II was measured and analyzed in this study. QT interval was defined as the interval between the first deflection of the QRS complex and the end of the T wave, which was measured using a tangent extending from the steepest section of the T wave downslope to where it crossed the T-P segment. All QT and RR intervals were averaged over three consecutive complexes in sinus rhythm and over all complexes on the 10-s lead II rhythm strip on the 12-lead ECGs in other rhythms.

The QTc intervals were calculated using Bazett’s formula (QTc = QT/√RR) [32, 33]. The ECG tracings were blinded and analyzed by two independent coders. Inter-reader discrepancies were resolved by direct comparison and adjudicated by another supervisor. A coefficient of reliability of 0.995 and Pearson’s correlation coefficient of 0.995 were calculated in the inter-reader reproducibility assessment for QT measurements. Comparisons of inter-reader QT measurements using the paired *t*-test did not reach statistical significance (*P* = 0.35). Extremely rapid (> 150 bpm) and extremely slow (< 40 bpm) heart rate recordings were excluded [34, 35]. In this study, QTc prolongation was categorized into three sex-specific categories as reported in a previous study [36], with cutoff values of ≤450 ms (normal), 451 to 470 ms (borderline), and > 470 ms (prolonged) for women, and ≤ 430 ms (normal), 431 to 450 ms (borderline), and > 450 ms (prolonged) for men.

### Statistical analysis

Data are expressed as mean ± SD or median and interquartile range (IQR). We used the Kolmogorov-Smirnov test to evaluate the normality of variables. Normally distributed variables were compared using one-way analysis of variance (ANOVA) with Tukey’s pairwise comparison. Before performing the statistical tests, we logarithmically transformed levels of serum TGs, creatinine, FABP4, and hs-CRP to achieve a normal distribution. Categorical variables are presented as frequency and/or percentages, and the χ^2^ test was used for inter-group comparisons. Multivariate logistic regression analysis using the existence of an abnormal QTc interval as a dependent variable was conducted to determine the relative contributions to the outcome variable made by each variable.

We also recorded the occurrence of major adverse cardiac events (MACEs) after the patients had been discharged from the hospital, defined as all-cause mortality or re-hospitalization for a repeat percutaneous coronary intervention (PCI) or coronary artery bypass grafting, or for a cardiovascular-related illness including HF, recurrent angina pectoris, and nonfatal reinfarction. We also divided the distribution of plasma FABP4 levels into tertiles as follows: first tertile, ≤ 7.4 ng/mL; second tertile, 7.5 to 16.25 ng/mL; and third tertile, > 16.25 ng/mL. Laboratory features and ECG data in each tertile were described and tested for trend across plasma FABP4 tertiles using linear regression analysis and the Cochran-Armitage trend test for categorical variables including MACEs, medications, and other diseases. Correlations among FABP4 and biochemical and anthropometric parameters were analyzed using Pearson’s correlation analysis, and associations among QTc interval and FABP4 level and eGFR were analyzed using linear regression analysis. All tests were two-tailed, and a *P*-value of < 0.05 was considered to be statistically significant. Statistical analysis was performed using JMP software version 7.0 for Windows (SAS Institute, Cary, NC, USA).

## Results

Table 1 presents the baseline clinical, angiographic and biochemical data of the participants according to QTc prolongation status. One hundred and 71patients were defined as having a normal QTc interval, 88 patients were defined as having borderline QTc prolongation, and 138 patients were defined as having an abnormal QTc interval. The abnormal QTc interval group had a significantly higher serum FABP4 level than the borderline and normal QTc interval groups (15.9 ng/mL [IQR 6.7 to 41.4] vs. 10.2 ng/mL [IQR 5.3 to 19.7] vs. 8.5 ng/mL [IQR 5.2 to 14.4], respectively, *P* < 0.0001). In addition, the abnormal QTc interval group were older (*P* = 0.003) and had higher prevalence rates of diabetes mellitus (*P* = 0.003), hypertension (*P* = 0.0002), and HF (*P* < 0.0001). Furthermore, the abnormal QTc interval group had a higher white blood cell count, higher levels of fasting glucose, HbA1c, creatinine, uric acid, and blood urea nitrogen, a lower prevalence rate of hyperlipidemia, and lower levels of sodium, albumin, hematocrit, and hemoglobin than the normal QTc interval group. Moreover, the abnormal QTc interval group (prolonged QT interval) had a higher hs-CRP level and a lower eGFR than the normal and borderline QTc interval groups. There were no significant differences in smoking status, body mass index (BMI), waist circumference, smoking status, SBP, DBP, levels of calcium, potassium, TGs, HDL-C, and LDL-C, total cholesterol, number of diseased coronary arteries, Gensini score, PCIs, number of stents, and receiving beta-blockers, diuretics, and statin therapy among the three groups. In addition, all of the participants had stable angina with a good thrombolysis in myocardial infarction (TIMI) flow grade. As a result, there were no significant deteriorations or differences between the TIMI flow grade before and after the coronary angiography or angioplasty. Moreover, because all of our electrocardiographic parameters were collected before the examination, we believe that changes in the TIMI flow grade would not affect our results and conclusions.

**Table 1:** Baseline characteristics of the study population stratified by category of QTc prolongation at baseline^a^

Variable	All	Normal	Borderline	Abnormal	*P*-value
No	397	171	88	138	
Sex (male/female)	308/89	122/49	74/14	112/26	0.030
Age (years)	66.7 ± 11.5	64.9 ± 11.8	66.1 ± 11.4	69.3 ± 11.0	0.003
Age range	37–99	37–99	39–93	40–94	
BMI (kg/m^2^)	25.9 ± 3.7	26.0 ± 3.6	26.1 ± 3.5	25.6 ± 3.9	0.510
Waist (cm)	91.4 ± 9.7	91.4 ± 10.0	93.0 ± 9.0	90.3 ± 9.6	0.228
Hypertension (n, %)	285 (71.8)	106 (62.0)	64 (72.7)	115 (83.3)	0.0002
Hyperlipidemia (n, %)	259 (65.2)	120 (70.2)	60 (68.2)	79 (57.3)	0.048
Diabetes mellitus (n, %)	166 (41.8)	55 (32.2)	43 (48.9)	68 (49.3)	0.003
Heart failure (n, %)	65 (16.4)	11 (6.4)	13 (14.8)	41 (29.7)	< 0.0001
Current smoking (n, %)	188 (47.4)	75 (43.9)	43 (48.9)	70 (50.7)	0.461
QTc interval (ms)	446 ± 37	416 ± 19	444 ± 9	485 ± 27	< 0.0001
Systolic blood pressure (mmHg)	133 ± 22	130 ± 19	133 ± 20	135 ± 26	0.190
Diastolic blood pressure (mmHg)	77 ± 14	77 ± 13	79 ± 14	77 ± 14	0.513
Sodium (mEq/L)	138.6 ± 4.0	139.1 ± 3.2	138.8 ± 3.6	137.9 ± 4.8	0.018
Potassium (mEq/L)	3.9 ± 0.7	3.9 ± 0.5	3.8 ± 0.6	4.0 ± 0.9	0.180
Calcium (mg/dl)	8.8 ± 0.9	8.9 ± 0.8	8.7 ± 0.7	8.7 ± 1.1	0.485
Fasting glucose (mg/dl)	146.8 ± 81.0	132.0 ± 60.2	145.1 ± 66.6	160.3 ± 81.7	0.002
HbA1c (%)	7.0 ± 1.8	6.8 ± 1.5	7.1 ± 1.5	7.3 ± 2.1	0.034
T-cholesterol (mg/dl)	179.7 ± 43.0	181.3 ± 39.4	180.6 ± 41.6	177.2 ± 48.1	0.701
Triglyceride (mg/dl)	121.0(89.0–172.0)	120.5(87.0–172.0)	122.5(89.0–182.8)	120.5(92.0–169.0)	0.764
HDL-cholesterol (mg/dl)	39.9 ± 13.4	41.3 ± 14.5	40.4 ± 14.8	37.9 ± 10.7	0.092
LDL-cholesterol (mg/dl)	104.7 ± 35.6	107.1 ± 34.2	104.1 ± 36.3	102.2 ± 36.7	0.482
Uric acid (mg/dl)	6.9 ± 2.0	6.5 ± 1.7	6.8 ± 2.0	7.3 ± 2.3	0.016
Blood urea nitrogen (mg/dl)	25.0 ± 18.8	21.5 ± 16.7	24.9 ± 18.6	29.0 ± 20.5	0.006
Creatinine (mg/dl)	1.2 (1.1–1.6)	1.2 (1.1–1.4)	1.2 (1.1–1.4)	1.3 (1.1–2.5)	< 0.0001
Albumin (g/dl)	3.9 ± 0.4	4.0 ± 0.4	3.9 ± 0.4	3.8 ± 0.4	< 0.0001
Hematocrit (%)	39.2 ± 6.6	40.6 ± 6.4	39.2 ± 6.2	37.6 ± 6.6	0.0003
Hemoglobin (g/dl)	13.1 ± 2.2	13.5 ± 2.1	13.2 ± 2.2	12.6 ± 2.3	0.002
Estimated GFR (ml/min/1.73m^2^)	56.3 ± 24.0	62.3 ± 20.3	57.6 ± 24.0	47.8 ± 26.1	< 0.0001
Fatty acid-binding protein 4 (ng/mL)	10.6 (6.0–22.1)	8.5 (5.2–14.4)	10.2 (5.3–19.7)	15.9 (6.7–41.4)	< 0.0001
Hs-CRP (mg/L)	2.2 (0.7–6.4)	1.5 (0.6–4.0)	2.3 (0.6–7.2)	4.5 (1.2–11.7)	< 0.0001
White blood cell count (× 10^9^/L)	8.468 ± 3.513	7.851 ± 2.951	8.481 ± 3.770	9.228 ± 3.847	0.003
No. of diseased coronary arteries	1.7 ± 1.1	1.6 ± 1.1	1.7 ± 1.1	1.8 ± 1.1	0.188
Gensini score	36.0 (15.5–80.5)	41.5 (20.0–85.5)	34.0 (12.0–64.0)	30.3 (15.0–88.4)	0.182
Percutaneous coronary intervention	323 (81.4)	133 (77.8)	73 (83.0)	117 (84.8)	0.264
Number of stent	0 (0–1)	0 (0–1)	0 (0–1)	0 (0–0.3)	0.495
Anti-arrhythmic medication (n, %)	113 (28.5)	43 (25.2)	35 (39.8)	35 (25.4)	0.029
Beta-blockers (n, %)	68 (17.1)	26 (15.2)	22 (25.0)	20 (14.5)	0.084
Diuretics (n, %)	28 (7.1)	8 (4.7)	7 (8.0)	13 (9.4)	0.252
Statins (n, %)	107 (27.0)	47 (27.5)	27 (30.7)	33 (23.9)	0.524

Data are expressed as mean ± SD, number (percentage), or median (interquartile range). *HDL* high-density lipoprotein, *LDL* low-density lipoprotein, *Hs-CRP* high-sensitivity C-reactive protein. ^a^Classification of QTc prolongation: normal men, ≤ 430 ms; women ≤450 ms; borderline men 431–450 ms; women 451–470 ms; abnormal men ≥451 ms; women ≥471 ms

Multivariate logistic regression analysis was then used to evaluate the effects of plasma FABP4 level and other risk factors for arrhythmia in the patients with an abnormal QTc interval. The results showed positive associations between an abnormal QTc interval and male sex and higher plasma FABP4 level (Table 2).

**Table 2:** Multiple logistic regression analysis with the presence of abnormal QTc interval as the dependent variable

	exp (B)	95% Confidence Interval	*P*-value
Age	1.02	0.99–1.05	0.238
Male sex	2.70	1.16–6.28	0.022
Body mass index	1.00	0.92–1.09	0.973
Systolic blood pressure	1.00	0.99–1.02	0.686
Fasting glucose	1.00	0.99–1.01	0.133
Total cholesterol	1.00	0.99–1.00	0.209
Estimated glomerular filtration rate	0.99	0.98–1.01	0.671
Sodium	0.97	0.90–1.04	0.373
Potassium	0.83	0.50–1.36	0.454
Calcium	0.84	0.58–1.22	0.362
Use of anti-arrhythmic drugs	0.63	0.24–1.64	0.341
Use of antihypertensive drugs	0.72	0.22–2.43	0.599
Fatty acid-binding protein 4	1.02	1.00–1.03	0.017

To determine the effects of FABP4 plasma level on the electrocardiographic parameters, we classified the patients into three groups according to the tertile of FABP4 plasma level as follows: first tertile ≤7.4 ng/mL, second tertile 7.5 to 16.25 ng/mL, and third tertile > 16.25 ng/mL. Significant trends were noted in the associations among FABP4 level and heart rate, left ventricular ejection fraction (LVEF), hs-CRP, QTc interval, QRS duration, hypertension, diabetes, HF, and CKD (*P* for trend < 0.05; Table 3). In the patients who did not have a MACE, the mean follow-up period was 32.6 months (maximum, 106 months). Nine (6.8%) of the patients in the highest FABP4 plasma tertile died of any cause, compared to one (0.8%) in the lowest tertile. Significant trends were noted among the associations between FABP4 level and MACEs, HF, and mortality (*P* for trend < 0.05; Table 3). However, no significant differences were found in coronary intervention results with quantitative angiographic analysis including minimal lumen diameter, final minimal lumen diameter, acute gain, percent stenosis, and reference lumen diameter among the three groups of plasma FABP4 (all *P* > 0.05).

**Table 3:** Prevalence of major adverse cardiovascular events and all-cause mortality and electrocardiographic parameters according to fatty acid-binding protein 4 level

Parameter	First tertile	Second tertile	Third tertile	*P* for trend
FABP4 (ng/mL)	≤7.4	7.5–16.25	> 16.25	
Number	132	133	132	
LVEF (%)	62.7 ± 11.5	62.9 ± 11.1	57.9 ± 12.8	0.001
Hs-CRP (mg/L)	1.3 (0.4–4.6)	1.8 (0.7–4.0)	4.4 (1.5–10.1)	0.043
White blood cell count (×10^9^/L)	8.212 ± 2.991	8.011 ± 3.322	9.188 ± 4.058	0.356
ECG parameters				
Heart Rate (bpm)	72.0 ± 16.8	74.1 ± 16.2	82.4 ± 16.6	0.001
PR interval (ms)	168.2 ± 34.0	166.4 ± 33.6	172.7 ± 32.4	0.812
QRS duration (ms)	95.1 ± 13.4	96.0 ± 18.9	99.5 ± 19.9	0.020
QT interval (ms)	403.1 ± 39.5	402.1 ± 38.2	399.9 ± 45.0	0.743
QTc interval (ms)	436.0 ± 30.7	440.2 ± 34.4	461.5 ± 39.2	< 0.0001
Medication (n, %)				
Anti-arrhythmic medication	34 (25.8)	38 (28.6)	41 (31.1)	0.634
Beta-blockers	22 (16.7)	23 (17.3)	23 (17.4)	0.985
Diuretics	12 (9.1)	6 (4.5)	10 (7.6)	0.333
Statins	38 (28.8)	36 (27.1)	33 (25.0)	0.786
Hypertension (n, %)	77 (58.3)	99 (74.4)	109 (82.6)	< 0.0001
Dyslipidemia (n, %)	84 (63.6)	92 (69.2)	83 (62.9)	0.501
Diabetes (n, %)	31 (23.5)	53 (39.9)	82 (62.1)	< 0.0001
Heart failure (n, %)	14 (10.6)	15 (11.3)	36 (27.3)	0.0002
Chronic kidney disease^a^ (n, %)	36 (27.3)	58 (43.6)	105 (79.6)	< 0.0001
MACEs (n, %)	41 (31.1)	47 (35.3)	66 (50.0)	0.004
All-cause Mortality (n, %)	1 (0.8)	4 (3.0)	9 (6.8)	0.026
Non-fatal outcome (n, %)				
Heart failure	6 (4.6)	6 (4.5)	15 (11.4)	0.028
Target lesion revascularization	28 (21.2)	32 (24.1)	31 (23.5)	0.562
Recurrent myocardial Infarction	6 (4.6)	5 (3.8)	11 (8.3)	0.180

Data are expressed as mean ± SD, number (percentage), or median (interquartile range). *FABP4* fatty acid-binding protein 4, *LVEF* left ventricular ejection fraction, *Hs-CRP* high-sensitivity C-reactive protein, *ECG* electrocardiography, *QTc* corrected QT, *MACEs* major adverse cardiovascular events. ^a^Chronic kidney disease was defined as an eGFR< 60 mL/min/1.73 m^2^

**Fig. 1:**
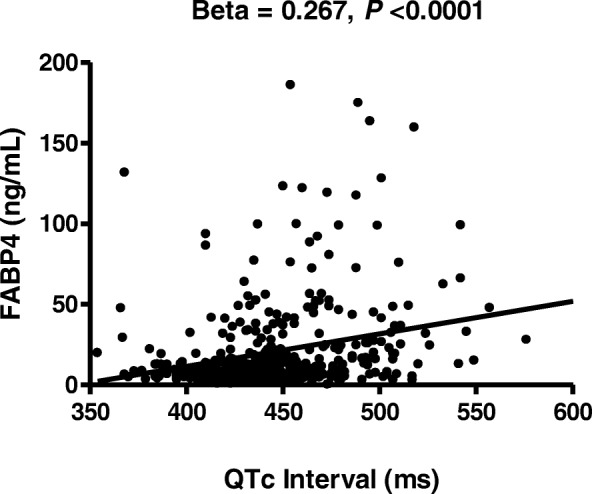
Association between the corrected QT (QTc) interval and plasma fatty acid-binding protein 4 (FABP4) concentration. The QTc interval was positively correlated with FABP4 concentration

A positive association was observed between FABP4 level and QTc interval (beta = 0.267, *P* < 0.0001; Fig. 1). Pearson’s correlation analysis revealed that FABP4 plasma level was positively correlated with age, SBP, DBP, levels of creatinine, fasting glucose, hs-CRP, HbA1c, and blood urea nitrogen, and QTc interval. In addition, eGFR, and levels of HDL-C, hemoglobin, and hematocrit were negatively correlated with levels of FABP4 (Table 4).

**Table 4:** Pearson’s correlation analysis of fatty acid-binding protein 4with clinical laboratory data

Variable	r	*P*-value
Age	0.113	0.025
Body mass index	0.043	0.391
Systolic blood pressure	0.201	0.0001
Diastolic blood pressure	0.124	0.014
QTc interval	0.267	< 0.0001
Fasting glucose	0.169	0.001
HbA1c	0.136	0.013
Total-cholesterol	−0.015	0.767
Triglycerides	0.080	0.111
HDL-cholesterol	−0.112	0.028
LDL-cholesterol	−0.094	0.062
Uric acid	0.106	0.084
Blood urea nitrogen	0.381	< 0.0001
Creatinine	0.589	< 0.0001
eGFR	−0.594	< 0.0001
Hemoglobin	−0.407	< 0.0001
Hematocrit	−0.400	< 0.0001
Hs-CRP	0.280	< 0.0001
White blood cell count	0.073	0.145
Current smoking	0.102	0.098

*HDL* high-density lipoprotein, *LDL* low-density lipoprotein, *eGFR* estimated glomerular filtration rate, *Hs-CRP* high-sensitivity C-reactive protein

## Discussion

In the current study, we found that plasma FABP4 levels were independently associated with abnormal QTc interval in patients with stable angina and CKD. The associations between plasma FABP4 and QTc prolongation still persisted after controlling for conventional risk factors including age, sex, SBP, BMI, use of anti-arrhythmic and antihypertensive medications, eGFR, and levels of potassium, sodium, calcium, fasting glucose, and total cholesterol. Furthermore, we also found that the level of hs-CRP was higher in the patients with QTc prolongation, and that hs-CRP was well correlated with FABP4 levels. These findings are in agreement with current evidence regarding the association between ventricular arrhythmias and inflammation [37, 38].

The secretion of FABP4 from visceral or subcutaneous adipose tissue, epicardial fat tissue, or macrophages can affect heart dysfunction through paracrine and endocrine pathways. The expression of FABP4 has been shown to be strongly induced during adipocyte differentiation [39], and thus several studies have proposed that this molecule can be used as a marker of adipocyte differentiation [40, 41]. Several studies have shown the induction of FABP4 expression when monocytes differentiate to macrophages in a manner similar to adipocytes, and a wide range of proinflammatory stimuli have been shown to regulate its expression in these cells [42, 43]. In addition, FABP4 has been reported to induce the formation of foam cells, increase the accumulation of cholesterol ester, and induce inflammatory responses in macrophages through activation of the JNK-AP-1 and IKK-NF-κB pathways [44, 45]. Our findings suggest that the inflammatory activity reflected by hs-CRP level was a strong etiologic factor for the higher levels of plasma FABP4 in the patients with QTc prolongation. FABP4 has recently been shown to be associated with an increased cardiometabolic risk, and previous studies have reported both clinical and experimental evidence that FABP4 is a relevant factor in atherosclerosis and CAD [24, 46]. Furthermore, it has been directly associated with cardiac alterations such as left ventricular hypertrophy and both systolic and diastolic cardiac dysfunction [47]. Despite the strong evidence showing the effect of plasma FABP4 concentration on cardiovascular diseases, the relationship between plasma FABP4 level and electrocardiographic parameters are unknown, especially its arrhythmogenic effect on the QTc interval.

Patients with CKD have been reported to have a high frequency of prolonged QTc interval [48]. Moreover, a prolonged QTc interval has been shown to be a marker of defective cardiac repolarization, and an important cause of sudden cardiac death and cardiac arrhythmias. The major factors contributing to repolarization defects include the administration of drugs, genetic defects, HF, diabetes mellitus, sex, renal failure, hypokalemia, and hypothyroidism. Repolarization defects have also been reported in the sudden death of athletes. However, the precise pathophysiological mechanisms behind a prolonged QT interval have yet to be elucidated. In particular, as renal function deteriorates, levels of pro-inflammatory cytokines increase [49]. Patients with CKD have been reported to have higher chronic inflammation, which could be another potential mechanism resulting in cardial fibrosis, vascular damage, sympathetic overactivity, and ion channel malfunction, which can increase the risk of cardiac arrhythmias and death [14, 50]. In addition, higher levels of markers of inflammation have been associated with the outcomes of patients with acute coronary syndromes and the risk of atherosclerotic complications [51]. Furthermore, previous studies have shown associations between CRP and cytokines such as IL-6 and atrial fibrillation and other arrhythmias in the absence of CAD [13], raising the possibility of a direct electrophysiological effect. In support of this hypothesis, the modulation of ion channel function and production of arrhythmias by cytokines such as platelet-activating factor have been described [12]. Consistent with these findings, our findings suggest that inflammatory activity as reflected by hs-CRP level was a strong etiologic factor for the higher levels of plasma FABP4 in the patients with QTc prolongation. In addition, the patients with higher levels of plasma FABP4 had a lower LVEF, higher prevalence of CKD, and higher hs-CRP level compared with the other groups. FABP4 has been associated with adiposity and metabolic disorders, and it has been reported to be a novel predictor of cardiovascular mortality in end-stage renal disease [52]. In addition, previous studies using animal models have indicated that FABP4 plays a significant role in several aspects of the metabolic syndrome, including insulin resistance, type 2 diabetes, and atherosclerosis, through its action at the interface of metabolic and inflammatory pathways in adipocytes and macrophages [46, 53–55]. The results of the present study support the idea [44–46] that FABP4 may act through an inflammation response to play an important role in the pathophysiology of QTc interval prolongation in patients with stable angina and CKD. Furthermore, in the present study, the patients with higher levels of plasma FABP4 also had higher rates of hypertension, diabetes, and HF, thereby raising the possibility that FABP4 directly contributes to cardiac repolarization defects and is associated with QTc prolongation.

We also found that there were significant trends in the associations among FABP4 level and MACEs and all-cause mortality. Moreover, we found that plasma FABP4 levels were most strongly positively correlated with blood urea nitrogen and creatinine. This suggests that the level of plasma FABP4 increases with the progression of renal failure. Furuhashi et al. suggested that the concentration of plasma FABP4 may be a marker of metabolic syndrome, and that it could also be used in patients with end-stage renal disease and as a novel predictor of cardiovascular mortality in patients at high risk of atherosclerotic cardiovascular events [52].

The current study has some limitations. First, as a cross-sectional study, we could not make a cause-and-effect conclusion between increased plasma FABP4 levels and QTc prolongation. Further long-term follow-up studies are needed to clarify the role of plasma FABP4 in QTc prolongation. Second, in our study, all the patients’ ECGs parameters including QT interval and QTc were measured by same medical technician using identical computer-based method, as a result, we did not evaluate the variability of measured QT interval. Third, in accordance with the guidelines of the Bureau of National Health Insurance, serum magnesium is not routinely measured in patients with stable angina, and thus magnesium data were not available in this study. Previous studies have reported that low serum magnesium has been linked to increased risk of atrial and ventricular arrhythmias and cardiovascular mortality [56, 57]. However, no association has been reported between serum magnesium levels and QTc interval [33]. Fourth, if the study population had different diseases (e.g. acute coronary syndrome and myocardial infarction), the diverse disease severity and condition of the study population may have impacted the results. To avoid selection bias, we chose individuals with stable angina for this study, thus the results of the present study might not be generalizable to other populations. Furthermore, in the present study, we found that an abnormal QTc interval was positively associated with male sex. Due to the small number of female patients we could not perform subgroup analysis by sex, and further studies are required to clarify this issue. Moreover, whether FABP4 is associated with the expression of cardiomyocyte electrophysiology, cardiac ion channels, and the underlying mechanisms are still unclear. Further future studies are warranted to elucidate these issues.

## Conclusion

The results of this study indicated that a prolonged QTc interval in patients with stable angina and CKD was correlated with an elevated FABP4 level. Further studies are required to investigate the associations between FABP4 level and QTc interval.

## Data Availability

Due to the regulations of our Institutional Research Ethics Committees and Institutional Review Boards, the detailed data of the study subjects cannot be disclosed publicly. However, the data set can be provided on request by any journal review board after approval from the Institutional Research Ethics Committees.

## Abbreviations

ANOVA: Analysis of variance
BMI: Body mass index
CAD: Coronary artery disease
CKD: Chronic kidney disease
CRP: C-reactive protein
DBP: Diastolic blood pressure
ECGs: Electrocardiograms
eGFR: estimated glomerular filtration rate
FABP4: Fatty acid binding protein 4
HDL-C: High density lipoprotein- cholesterol
HF: Heart failure
hs: high-sensitivity
IQR: Interquartile range
LDL-C: Low density lipoprotein- cholesterol
LVEF: Left ventricular ejection fraction
MACEs: Major adverse cardiac events
MLD: Minimal lumen diameter
QCA: Quantitative coronary angiography
QTc: Corrected QT
SBP: Systolic blood pressure
TGs: Triglycerides
